# Deciphering the role of endophytic microbiome in postharvest diseases management of fruits: Opportunity areas in commercial up-scale production

**DOI:** 10.3389/fpls.2022.1026575

**Published:** 2022-11-17

**Authors:** Madhuree Kumari, Kamal A. Qureshi, Mariusz Jaremko, James White, Sandeep Kumar Singh, Vijay Kumar Sharma, Kshitij Kumar Singh, Gustavo Santoyo, Gerardo Puopolo, Ajay Kumar

**Affiliations:** ^1^ Department of Biochemistry, Indian Institute of Science, Bengaluru, India; ^2^ Department of Pharmaceutics, Unaizah College of Pharmacy, Qassim University, Unaizah, Saudi Arabia; ^3^ Smart-Health Initiative (SHI) and Red Sea Research Center (R.S.R.C.), Division of Biological and Environmental Sciences and Engineering (B.E.S.E.), King Abdullah University of Science and Technology (K.A.U.S.T.), Thuwal, Saudi Arabia; ^4^ Department of Plant Biology, Rutgers University, The State University of New Jersey, New Brunswick, NJ, United States; ^5^ Division of Microbiology, Indian Council of Agricultural Research (ICAR), New Delhi, India; ^6^ Centre of Advanced Study in Botany, Banaras Hindu University, Varanasi, India; ^7^ Campus Law Centre, Faculty of Law, University of Delhi, New Delhi, India; ^8^ Instituto de Investigaciones Químico Biológicas, Universidad Michoacana de San Nicolás de Hidalgo, Morelia, Mexico; ^9^ Center Agriculture Food Environment, University of Trento, Trentino, TN, Italy

**Keywords:** endophytes, molecular interactions, biocontrol screening, commercial hurdles, postharvest management, fruits

## Abstract

As endophytes are widely distributed in the plant’s internal compartments and despite having enormous potential as a biocontrol agent against postharvest diseases of fruits, the fruit–endophyte–pathogen interactions have not been studied detail. Therefore, this review aims to briefly discuss the colonization patterns of endophytes and pathogens in the host tissue, the diversity and distribution patterns of endophytes in the carposphere of fruits, and host–endophyte–pathogen interactions and the molecular mechanism of the endophytic microbiome in postharvest disease management in fruits. Postharvest loss management is one of the major concerns of the current century. It is considered a critical challenge to food security for the rising global population. However, to manage the postharvest loss, still, a large population relies on chemical fungicides, which affect food quality and are hazardous to health and the surrounding environment. However, the scientific community has searched for alternatives for the last two decades. In this context, endophytic microorganisms have emerged as an economical, sustainable, and viable option to manage postharvest pathogens with integral colonization properties and eliciting a defense response against pathogens. This review extensively summarizes recent developments in endophytic interactions with harvested fruits and pathogens—the multiple biocontrol traits of endophytes and colonization and diversity patterns of endophytes. In addition, the upscale commercial production of endophytes for postharvest disease treatment is discussed.

## Introduction

In the recent era of climate change and the rising global population, food security is one of the most critical issues worldwide. At the same time, postharvest losses of fresh products, including fruits, vegetables, or horticultural crops, accelerate food security challenges. Currently, it has been estimated that approximately 50%–60% of the total agricultural production (Kumar and Kalita, 2017) and 30%–50% of the total fruit production are lost after harvesting due to improper storage, attack of pathogens, or the incidence of diseases (Zhang et al., 2017). However, on the broad industrial scale or even a laboratory scale, various chemical pesticides or fungicides have been broadly employed to prevent postharvest loss caused by phytopathogens or diseases. Nevertheless, the undistributed use of chemical pesticides adversely affects the nutrient constituents, texture, flavor, and quality of the fruits and negatively impacts consumer health. Furthermore, the emergence of resistant pathogen varieties against existing pesticides is a severe problem (Hahn, 2014; Nicolopoulou-Stamati et al., 2016). Therefore, the negative consequences of chemical pesticides on fruit quality, human health, and the environment urgently need the development of a reliable and sustainable approach to replace toxic agrochemicals with suitable microbial antagonists.

Utilizing the endophytic microbiome as a biocontrol agent (BCA) during preharvest or postharvest storage conditions has emerged as a suitable alternative to chemical pesticides in the last few years (Singh et al., 2019; Kumar et al., 2021; Ahmad et al., 2022). Endophytes are the microbes that colonize intercellular/intracellular spaces of plants without causing any apparent sign of infection (Bacon and White, 2016; Pathak et al., 2022). Endophytes are well known for inducing plant growth-promoting traits and ameliorating biotic and abiotic stresses (Glassner et al., 2015). In addition, it synthesizes a plethora of bioactive compounds that enhance the host’s immune response and protect the plant from pathogen attacks or disease incidence (Nair and Padmavathy, 2014; Singh et al., 2017). For practical biocontrol efficacy, the most challenging task is the administration and establishment of microorganisms inside the host plant. An endophytic microbiome is a suitable option in this context due to better colonization and proliferation efficacy (Busby et al., 2016; O’Brien, 2017). Nevertheless, there is still a need to explore the endophytic microbiome for its practical application as microbial antagonistic agents against various phytopathogens or plant diseases during postharvest storage conditions.

Furthermore, the diversity of endophytic microbiome in the fruits, its role in biotic stress amelioration, and an insight into the mechanistic aspects are still under investigation (Aiello et al., 2019; Chaouachi et al., 2021). Therefore, research on the endophytic microbiome and its role in minimizing postharvest loss of horticultural crops, including fruits, needs special attention with an in-depth discussion regarding their prospects and their transition from lab to field or industry. This review summarizes the molecular interaction of plant endophytes, the diversity of endophytic microbiome, the screening of BCAs, and the technological aspect of endophytic microbiome postharvest management. This review also focuses on the literature and discussion on the modes of application, the future aspects, and the hurdles to be overcome for converting endophytes into the success stories of postharvest management of fruits in a sustainable manner.

## An overview of microbial endophytes

Plants host diverse communities of microorganisms as epiphytes (on the surface) or endophytes (inside the plant tissue) and share a complex relationship. These host–microbe interactions play significant roles in maintaining the plant normal physiology under biotic and abiotic stress conditions (Khalaf and Raizada, 2018; Verma et al., 2021). The term endophyte was firstly introduced by De Bary (1866) as the fungal species living inside the host tissue. However, Petrini (1991) considered endophytes, of either fungal or bacterial strains, as those that reside in the host tissue or plant for at least some part of their life cycle without causing any disease or apparent sign of infection. With technological advancement or next-generation sequencing (NGS), it has been estimated that each plant species harbors multiple endophytic microbes during its life cycle (Senthilkumar et al., 2011; Verma et al., 2021). The latest NGS revealed that *Proteobacteria* is the most prominent endophytic bacterial phylum, followed by *Actinobacteria*, *Firmicutes*, and *Bacteroidetes*. In contrast, Glomeromycota is the major fungal phylum followed by *Ascomycota* and *Basidiomycota*; however, *Pseudomonas*, *Pantoea*, *Acinetobacter*, and *Enterobacter* members of Gamma-Proteobacteria are the commonly found bacterial genera. Arbuscular mycorrhizal fungi (AMF) are the most prominent fungal taxa among endophytic fungi in plant tissues (Hardoim et al., 2015; Kumar et al., 2020; Verma et al., 2021).

The endophytic microbes within plant tissue interact with plants and modulate the plant’s growth, fitness, and physiology. The mutualistic endophytes live inside the host and mutually benefit each other; for example, endophytes produce phytohormones, solubilize nutrients, and modulate bioactive compounds of the host, all resulting in the growth and development of the plant, and in return, the plant provides shelter and nutrients to the endophytes (Papik et al., 2020; Khalaf and Raizada, 2020).

### Colonization by microbial endophytes

The host–endophyte share a complex relationship that is driven by various intrinsic and extrinsic factors (White et al., 2019; White et al., 2021). However, the entry or establishment of microorganisms in the host tissue is the primary step for any strain to be an endophyte (White et al., 2019; Micci et al., 2022). According to Kandel et al. (2017), endophytic colonization refers to the entry, growth, and multiplication of endophytes within the internal compartments of the plant host. However, colonization is a complex process regulated by different signaling molecules in several consecutive steps (Kumar et al., 2020). Firstly, the plant species attract the microbes by the specific components of their exudates, which are generally composed of sugars, organic acids, amino acids, lipopolysaccharides (LPSs), flavonoids, and proteins and may be specific for each microbial strain (White et al., 2019). The microbes showed a chemotactic response toward the specific components of the exudates and facilitated effective colonization (Oku et al., 2012). The motility of the microbial strain/s toward the host surface is facilitated by appendages that protrude from the cell surface, such as flagella, or through type IV pili (Knights et al., 2021). Several reports reinforce the importance of lateral appendages during this movement (Sauer and Camper, 2001; Zheng et al., 2015). For instance, flagella were reported to have direct involvement in adhering to *Azospirillum brasilense* with wheat roots (Pinski et al., 2019). Böhm et al. (2007) reported type IV pili and their direct role in the colonization of *Azoarcus* sp. BH72 to the surface and root interior of rice. However, attachment of the endophyte on the host surface is facilitated through secretory products such as exopolysaccharides (EPSs), LPSs, cell surface saccharides, and cellulase of the microbial strain. For example, Meneses et al. (2011) reported that the inactivation of gene *gumD*, which is responsible for EPS synthesis, decreased the colonization rate of the endophytic strain *Gluconacetobacter diazotrophicus* in rice roots.

Similarly, Monteiro et al. (2012) observed that inactivation of gene wssD, bcsZ, which are responsible for the synthesis of beta-1,4, glucanase (cellulose), decreased the colonization rate of *Herbaspirillum rubrisubalbicans* M1 in *Zea mays*. The endophytic microorganism, before its entry or colonization, confronts the challenges of oxidative environments of the host tissue. This situation is similar to the one the pathogens face during infection of the host. The host plant provides a barrier to oxidative burst, resulting in only a few microorganisms that can enter plant cells (White et al., 2019; White et al., 2021). Experiments have shown that this initial oxidative burst can be reduced by treating seedlings with low concentrations of humic substances, resulting in increased entry of bacteria into root cells at root tips (White et al., 2021). To be an endophyte, microbial strains must be able to survive in the oxidative environment within plant cells (Di Pietro and Talbot, 2017; White et al., 2019). In this context, several authors reported the successful acclimation potential of endophytic strains; for example, *Enterobacter* spp. encodes antioxidant enzymes during the colonization of poplar plants (Balsanelli et al., 2016).

Additionally, Malfanova et al. (2013) reported genes responsible for antioxidative enzymes used by *Klebsiella* to protect the host plant from reactive oxygen species (ROS). Similarly, strain *G. diazotrophicus* showed the expression of antioxidant enzyme genes during the early stage of colonization in rice plants (Meneses et al., 2017). In addition, the colonization efficacy of the endophyte depends upon several factors; host genotype, nutrient status, and specificity of microbial strain are the prime factors (Hardoim et al., 2015).

### Colonization patterns of endophytes and pathogens in the host tissue

**Figure 1:**
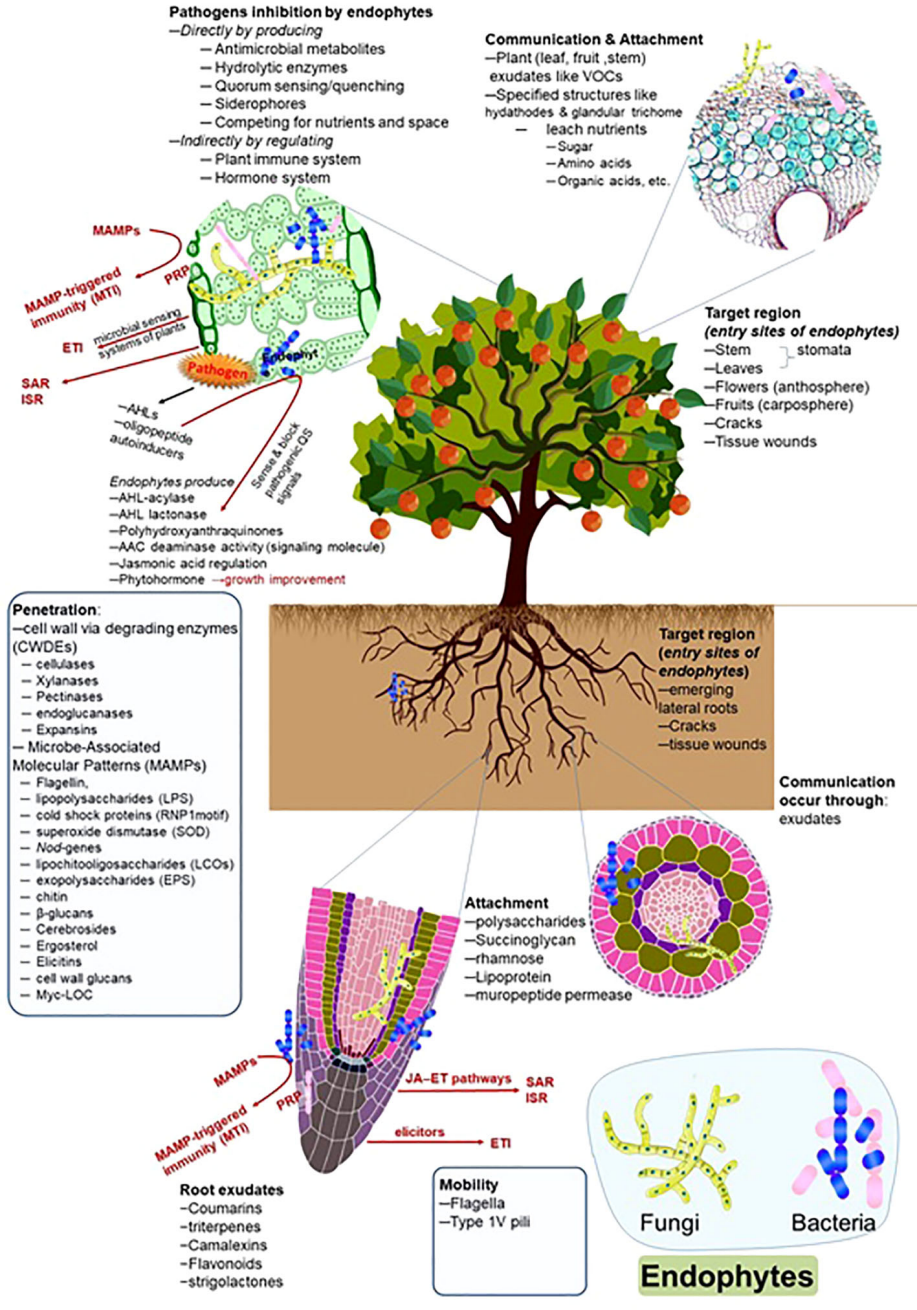
Endophytes and their interaction with the host plants. The figure describes the detailed role and approach of root exudates, communication, mobility, attachment, penetration, and target region (entry site) during endophyte colonization.

The colonization patterns of the pathogens and endophytes are similar to some extent. However, the response of plant defense systems differs and depends upon the nature of the microorganisms. Similarly, the expression patterns against oxidative stress are also different. Chen et al. (2020a) reported the colonization patterns of endophytic strain *Azoarcus olearius* and the pathogen *Xanthomonas oryzae* in rice plants and observed differential expression patterns of genes. The pathogen followed the salicylate pathway; however, the *Azoarcus* used the jasmonate signaling pathway during colonization. The colonization patterns of symbiotic endophytes and pathogenic strains are also dissimilar regarding secretions. Pathogenic strains secrete comparatively higher amounts of cell wall-degrading enzymes at the infection sites. In contrast, a lower amount of cell wall-degrading enzymes was reported during endophyte colonization, which could not elicit the plant immune system and make easy access to endophytes inside the host tissue (Elbeltagy et al., 2000; Reinhold-Hurek et al., 2006; Naveed et al., 2014). The overview of endophytic dynamics, entry, colonization, transmission, and interacted factors is presented in **Figure 1**.

### Diversity of endophytic microbiota in the fruit

The physiology and biochemistry of the plant depend upon the surrounding biotic and abiotic factors, which ultimately affect the diversity and composition of the microbiota, either epiphytes or endophytes. For instance, seasonal variations affect the number of plant exudates, which are a determining factor in rhizospheric microbial population and endophytic colonization (Wang et al., 2009; Kuffner et al., 2012). The genotype (Mocali et al., 2003), cultivars (Pettersson and Bååth, 2003), and host plant’s age influence endophytic microbial compositions.

Recently published reports reinforce the variation in the endophytic populations among the plant organs. For example, Ren et al. (2019a) reported variations in the endophytic bacterial microbiome among the different organs of the same Jingbai pear (*Pyrus ussuriensi* Maxim.) plant. Maximum richness and diversity were observed in the root tissue, followed by flower, stem, and fruit, and the lowest were in the leaf tissue. This report illustrates that each plant organ has a specific richness or diversity.

Furthermore, in another study, Ren et al. (2019b) reported variations in fungal richness or diversity in the different plant organs of the Jingbai pear forest. They observed that the root tissue had maximum fungal richness and diversity, followed by stem, fruit, and leaf, and the lowest were observed in the flower tissue. Thus, the diversity patterns of both bacteria and fungi are different in the same plants. Finally, Dong et al. (2019) reported a similar observation of bacterial distribution patterns among the root zone, rhizosphere, phyllosphere, and endosphere of roots, stems, leaves, fruits, and seeds of tomatoes under greenhouse conditions. They observed that the root zone and rhizospheric soil had the highest diversity and richness, followed by stem, flowers, and fruits; however, the lowest diversity and richness were observed in the phyllosphere tissue.

Abdelfattah et al. (2015) also reported that leaves contain higher diversity than flowers or olive fruits (*Olea europaea*), and the fungal diversity consequentially decreased from fruitlets to mature stages of the olive. However, the trends of the fungal community were very similar from fruitlets to the flowering stage, which later changed. However, the microbial diversity in the flower or fruit section is similar to the diversity of some other parts. Therefore, the uniqueness and diversity of endophytic microbiota may vary among the different compartments of the fruits (Ottesen et al., 2013). The uniqueness may be due to the ovaries, which turn into flesh and create a new environment that harbors specific microbiota or microbial strains (Tadych et al., 2012; Aleklett et al., 2014).

## Host–endophyte interaction in terms of biocontrol agents

It is well known that during plant–microbe interactions, microbial strains showed neutral, commensalism, mutualistic, or pathogenic interaction with the host plants. The establishment depends upon several factors, including the genotype of microorganisms or host plants and the surrounding environment (Brader et al., 2017). Plants rely on their sophisticated defense systems to counteract attacks of phytopathogens (Jones and Dangl, 2006), as the pathogenic strains secrete numerous biomolecules inside the host during infection. The host plant responds accordingly after recognizing conserved structure and elicits its immune behavior as the first line of defense to control the pathogen by the present pattern recognition receptors (PRRs). The PRRs sense the nature of microbes through the perception of microbe-associated molecular patterns (MAMPs) or pathogen-associated molecular patterns (PAMPs) (Plett and Martin, 2018). Bacterial flagellin, elongation factor Tu (EF-Tu), fungal chitin, and yeast mannans are the most commonly reported PAMPs/MAMPs (Newman et al., 2013).

**Figure 2:**
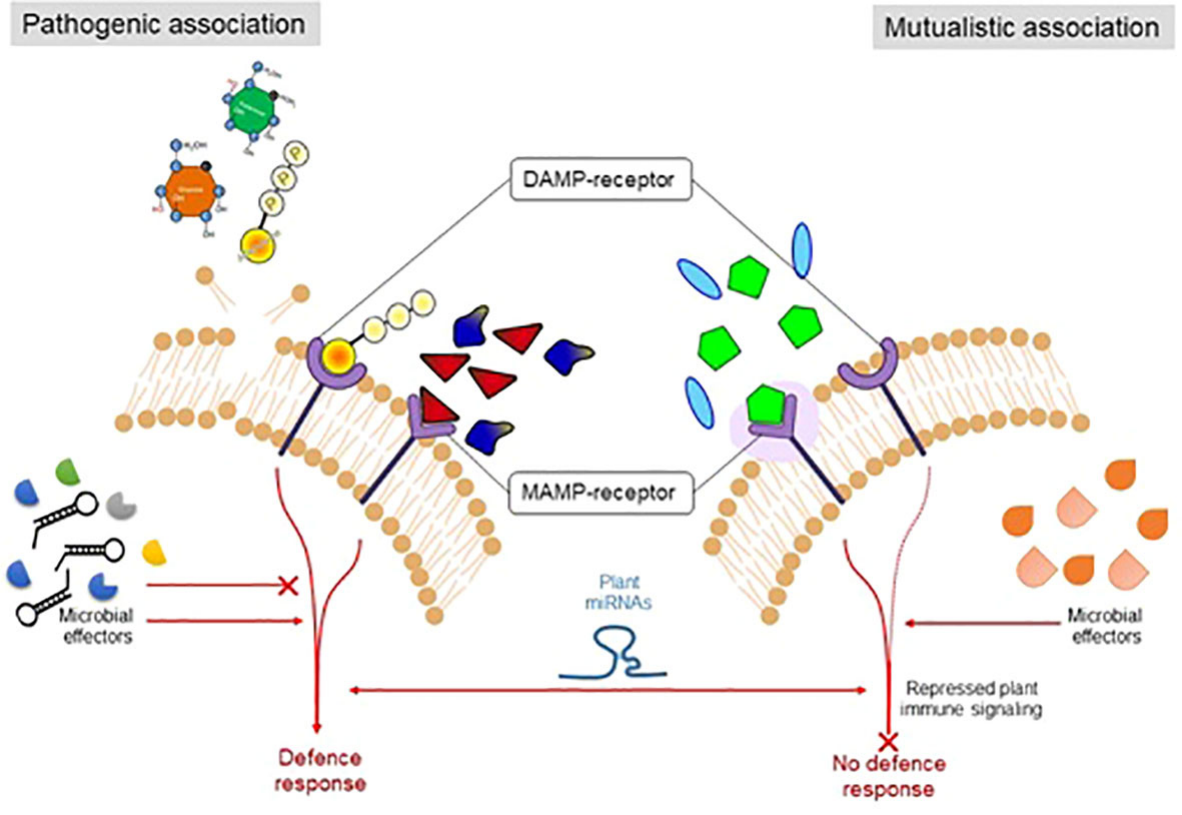
The figure illustrates the mechanism by which plants sense to differentiate symbiotic and pathogenic microorganisms.

During co-evolution with the host plant, pathogenic strains improved the strategies to suppress the MAMP/PAMP-triggered immunity. In response, the host plant developed a second line of defense known as effector-triggered immunity. The plant system develops receptors that sense or recognize the pathogen’s constituents. For instance, for the pathogenic microbes (biotrophic) that depend upon the nutrient uptake of living cells, a hypersensitive response may be activated, which leads to the programmed cell death of plants under attack (de Wit, 2007). However, this response must be suppressed in the case of necrotrophic pathogens or endophytes or symbiotic microorganisms (Liu et al., 2017). However, to cope with the plant immune system, the endophytic microorganisms produce their MAMPs, which do not significantly elicit the host immune or defense system. However, there is significant variation between the cell surface components (flagellin proteins in the endophytic microbes) of endophytic/symbiotic or pathogenic microbial strains (Trdá et al., 2014), which show differential patterns at the time of recognition by the receptors **(** **Figure 2** **)**.

## Endophytes as biocontrol agents

To explore endophytes as biological control agents, several factors have been considered relevant, including survival, stability, storage, application, and marketability. Despite the massive exploration of various microbial strains as BCAs *in vivo* or *in vitro*, only a limited number of strain/s, bacteria, fungi, or yeast, have been commercialized, and the possible reason is the survivability or stability of BCAs. The endospore formation of *Bacillus subtilis* or chlamydospore structure of *Trichoderma* makes them most suitable compared to other microbial strains because of stability or survivability under unfavorable conditions to fulfill the requirement of commercial exploitation. However, the endophytic microbiome can easily be administered, penetrating and colonizing the host tissue, unlike other microorganisms where colonization is a complex process. However, the effectiveness of BCAs against the pathogen may also depend upon various factors, including the growth or physiological state of the plant, genotype, colonization pattern, population dynamics, and the surrounding environmental conditions (Card et al., 2016; Bolívar-Anillo et al., 2020).

Recent studies have reported the antagonistic activities of a diverse range of endophytes, which is present on the fruit surface. A number of bacterial, actinomycetes, and fungal species are present on the fruit surface that can impact the growth of postharvest pathogens (Huang et al., 2021). Similar to field conditions, *Pseudomonas*, *Citrobacter*, *Paenibacillus*, *Burkholderia*, and *Bacillus* sp. are some of the most prevalent biocontrol bacteria found on fruit surfaces (Shi et al., 2013; Huang et al., 2021). The use of endophytic yeast *Metschnikowia pulcherrima* along with chitosan prevented the growth of *Alternaria alternata* in table grapes (Stocco et al., 2019). *Aureobasidium pullulans* prevented the growth of *Botrytis cinerea* and *Monilinia laxa* in sweet cherries and table grapes, decreasing the decomposition rate of fruits between 10% and 100% (Schena et al., 2003). *Pantoea dispersa* controlled the black rot of sweet potato by exhibiting antibiosis (Jiang et al., 2019). *Trichoderma* and *Nodulisporium* are some of the most found fungal BCAs on the carposphere. Recently, mycofumigation with the fungal volatile organic compounds (VOCs) has also gained attention to inhibit the growth of postharvest pathogens (Zhi-Lin et al., 2012). Suwannarach et al. (2013) reported on biofumigation with the *Nodulisporium* spp. CMU-UPE34, an endophytic fungus, to prevent the postharvest decay of citrus fruits. The endophytic fungal stain *Nodulisporium* sp. strain GS4d2II1 produced six different VOCs, which inhibited *Fusarium oxysporum* growth in cherry tomato fruits after their harvest (Medina-Romero et al., 2017). Details of endophytic microbial strains and their utilization in postharvest disease or pathogen control of fruits have been discussed in **Table 1**.

**Table 1:** Endophytic microbial strains used for the postharvest disease or pathogen management in fruits.

Endophytic strains	Domain	Disease/Pathogens	Plants/Fruits	References
***Bacillus velezensis* QSE-21**	Bacteria	Postharvest gray mold of fruit	Tomato	Xu et al., 2021
***Paenibacillus polymyxa***	Bacteria	*Penicillium digitatum*	Citrus	Lai et al., 2012
***Bacillus subtilis* L1-21**	Bacteria	*Penicillium digitatum*	Citrus Fruits	Li et al., 2022
**Endophytic bacteria**	Bacteria	*Monilinia laxa* and *Rhizopus stolonifer*	Stone fruits	Pratella et al., 1993
***Bacillus amyloliquefacies***	Bacteria	*Botryosphaeria dothidea*	Kiwi fruit	Pang et al., 2021
***Pseudomonas synxantha* **	Bacteria	Monilinia fructicola and *Monilinia fructigena*,	Stone fruit	Aiello et al., 2019
Lactobacillus plantarum CM-3	Bacteria	*Botrytis cinerea*	Strawberry fruit	Chen et al., 2020b
***Bacillus subtilis* L1-21**	Bacteria	Botrytis cinerea	Tomato	Bu et al., 2021
***Penicillium* sp.**	Fungi	*Botrytis cinerea*	Grapes fruits	Noumeur et al., 2015
***Daldinia eschscholtzii***	Fungi	*Colletotrichum acutatum*	Strawberry fruits	Khruengsai et al., 2021
***Saccharomycopsis fibuligera***	Yeast	Botrytis cinerea	Guava fruits	Abdel-Rahim and Abo-Elyousr, 2017
***Muscodor suthepensis CMU-Cib462***	Fungi	*Penicillium digitatum*	Tangerine fruit	Suwannarach et al., 2016
***Fusarium* sp.**	Fungi	*Fusarium oxysporum*, *Aspergillus niger* and *Rhizopus stolonife*	Postharvest pathogens of vegetables	Tayung et al., 2010

### Screening of endophytic biocontrol agents

The search for endophyte agents with biocontrol capacities is imperative in detecting those agents with excellent antagonistic capacities against potential pathogens. Detecting these characteristics depends on having better chances of generating microbial endophyte-based biocontrols with good chances of being successful in open field application and not just showing good actions in the laboratory. Next, we detail some tools for detecting and selecting endophytic BCAs. Screening microbial antagonists against various phytopathogens is one of the most crucial steps. The BCAs are generally screened on the basis of some specific characteristics such as parasitism, in which BCAs live together with the host plant, resulting in antagonistic effects (Mukherjee et al., 2012). Furthermore, strains having the capability to synthesize antimicrobial or volatiles compounds and enzymes such as pectinases and cutinases, which can interfere with pathogenicity factors or reduce the virulence of pathogens, are preferred for BCA screening (Zimand et al., 1996; Kapat et al., 1998).

However, other direct or indirect mechanisms have been employed to screen suitable BCAs for particular or broad-scale phytopathogens causing plant diseases. Dual-culture assay is one of the standard phenotype-based direct screening methods for identifying microbial antagonists during *in vitro* identification. In this assay, BCAs and pathogens were cocultivated on semisolid media. The pathogen’s antagonistic behavior toward BCAs and pathogenicity are evaluated by measuring the lesion diameter (Shi et al., 2014). During the evaluation, both the BCAs and the pathogen were grown together on the plates at different locations, and a significant decrease in mycelium growth and fungal spores was observed (Comby et al., 2017). In another case, the pathogen has been evenly spread over the plate, and BCA was spotted over the medium. The clear zone around the spotted BCA was measured to evaluate biocontrol activity. The larger the clear zone, the higher the biocontrol potential (Shehata et al., 2016).

Synthesis of antimicrobial compunds, either diffusible or volatile, by the microbial endophytic strain is also one of the parameters for biocontrol screening. During *in vitro* volatile analysis, the BCA and the pathogen grow on an agar base plate, which is grown under physically separated conditions and sealed with parafilm or tape to avoid VOC escape (Stinson et al., 2003). However, screening of BCAs in liquid media has also been done under which both the BCAs and pathogen were grown either simultaneously or consecutively, and their impact has been evaluated either by measuring the optical density or by the microscopic evaluation of pathogen spore or germination tube of mycelia tube (Omar and Abd-Alla, 1998).

However, *in vivo* screening is the standard method for evaluating potential BCAs under natural or greenhouse conditions through several parameters such as measuring lesion diameter, disease severity, or defined disease index (Lecomte et al., 2016). *In vivo* screening not only is based on antagonistic activity but also includes the physiological status of the plant by measuring water status (e.g., transpiration, stomatal conductance), variation in antioxidant activity (e.g., enzymatic activity levels), production of plant defense molecules (e.g., phytoalexins), morphological growth parameters such as plant height, the dry or fresh weight of certain plant parts, or the flowering date (Lecomte et al., 2016). The antagonistic potential of the BCAs varies with plant genotype or species; differences in host genotypes differentially regulate the physiological functions that may modulate the rate of infections and response of host immune systems. Similarly, the colonization potential of the endophytes, which depends upon the various physiochemical nature of plant exudates, also impacts the biocontrol potential against the pathogen more efficiently and effectively (Martin et al., 2015).

## Postharvest factors that affect the quality of food and disease incidence

Postharvest diseases can result from incorrect postharvest practices and faulty preharvest management. The significant postharvest factors that affect the storage of food are as follows.

### Fruit storage conditions

Fruits are generally transported to supermarkets and cold chains before reaching customers’ hands. Temperature, pH, and humidity conditions in cold chains significantly affect the growth of pathogens and endophytes (Carmona-Hernandez et al., 2019). Low pH due to fruit metabolism and high humidity support the growth of fungal pathogens (Arah et al., 2015). In addition, temperature and pH conditions also influence the production of volatile secondary metabolites (VOCs) from the microbes (Lazazzara et al., 2017; Fadiji and Babalola, 2020). In a study, a lower pH condition of the fermentation medium significantly influenced the production of phloroglucinol and gallic acid from isolated endophytic fungus *Colletotrichum gloeosporioides* (Gasong and Tjandrawinata, 2016).

### Physical handling and gaseous treatments

The rough handling of already ripened fruits invites the attack of pathogens on soft and brushed surfaces. In addition, mechanical injuries to the fruits due to improper handling can increase the metabolism and ethylene production, which can cause adverse biotic stresses on the stored fruits (Miller, 2003). The stored fruit’s carbon monoxide (CO) treatment increases ripening and decreases pathogen infestation. The *Alternaria* rot in jujube fruits was effectively controlled by CO application in fruit storage conditions (Zhang et al., 2020). High carbon dioxide concentration around fruits also reduced the respiratory activities and consumption of soluble solids, which results in a reduction in pathogen infection (Huyskens-Keil and Herppich 2013). Apart from the growth of pathogens, physical handling and food storage conditions can also play a significant role in the growth and secondary metabolite production of endophytes.

## Postharvest management strategies by endophytes: Action mechanisms

Endophytes are known to show a myriad of mechanisms against pathogens ranging from direct competition to change in the molecular architecture of the host plants. Endophytes against postharvest pathogens, being a relatively new field, require an in-depth literature review to understand the possible mechanisms employed against postharvest pathogens. Following are the possible mechanisms that endophytes employ to combat pathogenic attacks on the harvested fruits.

### Direct competition for space and nutrients

In the tripartite system of fruit–pathogen–endophyte interaction, the nutrition and space of the host are limited. Nitrogen, carbon, macronutrients, and micronutrients are essential for the survival of both endophytes and pathogens (Kumari et al., 2020a, b). Endophytes, being fast in growth and colonization, quickly occupy the exposed fruit surface and outnumber pathogens in the space competition and utilization of nutritional resources (Adame-Álvarez et al., 2014; Spadaro and Droby, 2016). Different studies have demonstrated the utilization of carbon resources by endophytic *Bacillus* spp., inhibiting spore germination of the pathogens; however, bacterial dosage needs to be optimized according to the fruit (Carmona-Hernandez et al., 2019). A phenotypic and gene transcription study revealed the increased expression of genes involved in nutrition uptake by the bacterium *Lactobacillus plantarum* when cocultivated with the pathogen *Aspergillus carbonarius* isolated from grape berries (Lappa et al., 2018). The *L. plantarum* culture effectively inhibited the growth of four fungal pathogens isolated from the grape berries. A 32%–90% inhibition in mycotoxin produced by *A. carbonarius* was also observed after coculturing with *L. plantarum*. Successful *in vivo* application of this bacterium not only may help in controlling postharvest pathogens but also will act as a source of probiotics for modulating gut microflora.

### Production of siderophores (iron-chelating compounds)

Iron is one of the essential minerals required for the growth, survival, and virulence of pathogens. Siderophores are the secondary microbial metabolites produced by many endophytes, which can form a tight and stable octahedral Fe(H2O6)3+ complex with available iron (Miethke and Marahiel, 2007). The exposed fruit surface is an adverse niche, where the bioavailability of nutrients, especially iron, is relatively low. In the competition for survival, endophytes are known to colonize faster than pathogens, chelating the available iron by producing several types of siderophores and thus depriving the postharvest pathogen of any iron source (Chowdappa et al., 2020). Genome mining of the endophytic *Pseudomonas fluorescens* BRZ63 has revealed siderophore production by the bacterium, protecting against several postharvest pathogens, including *Colletotrichum dematium* K, *Sclerotinia sclerotiorum* K2291, and *Fusarium avenaceum* (Chlebek et al., 2020). Many endophytic *Bacillus* sp. produce bacilibactin type of siderophore-protecting bacterial wilt in banana (Carmona-Hernandez et al., 2019). *Trichoderma* spp. has been known to produce hydroxamate siderophore, which can deplete iron and inhibit the growth of postharvest pathogens in apples and citrus fruits (Sood et al., 2020). Though the endophytic *Trichoderma* spp. is still in the nascent stage for controlling postharvest diseases of fruits, it can pave a new and sustainable path for the disease control of fruits after harvest. However, optimizing the concentration of endophytes and factors affecting siderophore production should not be neglected to increase endophytic efficiency against postharvest pathogens.

### Production of bioactive antimicrobial compounds and antibiosis

Endophytic microbiomes have recently emerged as potent and novel sources of secondary metabolites, many of which are antimicrobial. They are known to produce alkaloids, flavonoids, phenolics, terpenoids, steroids, non-ribosomal peptides, and VOCs (Kumari et al., 2018). For example, endophytic *Trichoderma* sp. produced antifungal epipolythiodioxopiperazines, peptaibols, koninginins, and pyrenes, which combat postharvest diseases in kiwi fruit, apple, and banana (Khan et al., 2020). The recently published review article by Huang et al. (2021) briefly covered the bioactive compounds produced by endophytes and how they enhance the resistance against postharvest diseases of fruit and vegetables. Similarly, Carmona-Hernandez et al. (2019) also covered the bioactive compounds, volatiles produced by the endophytic strains, and their role in postharvest disease management. The details of bioactive metabolites produced by endophytes, which can potentially be used against postharvest pathogens of fruits, are described in **Table 2**.

**Table 2:** Bioactive compounds produced by endophytic microbes used in the management of postharvest diseases of fruits.

Endophytic microbes	Production of bioactive compound	Putative role against postharvest pathogens	References
***Bacillus subtilis***	Iturin A, lipopolysaccharide	Antifungal activity against *F. oxysporum*, *Pythium ultimum*, and *Phytophthora* sp.	Ek-Ramos et al., 2019
***Bacillus* sp.**	Surfactin, fengycin	Used against bacterial diseases	Jasim et al., 2016
***Pseudomonas aeruginosa***	Phenyltetradeca-2,5-dienoate	Used against bacterial diseases	Pratiwi et al., 2017
***Bacillus amyloliquefaciens* CEIZ-11**	lipopolysaccharide	Antifungal activity against *Botrytis cinerea* and Alternaria alternata	Zouari et al., 2016
***Pseudomonas putida* BP25**	VOCs	Antifungal activities against *Phytophthora capsici* and *Radopholus similis*	Sheoran et al., 2015
***Chaetomium globosum***	Chaetomugilin A and D	Antifungal activity against *Fusarium* sp. and *Verticillium* sp.	Pimentel et al., 2011
***Trichoderma lixii (IIIM-B4)***	Peptaibol	Shows antibacterial activities	Katoch et al., 2019
***Trichoderma* sp.**	VOCs	Antifungal activities against *Sclerotium rolfsii* and *Fusarium oxysporum*	Rajani et al., 2021
***Aspergillus fumigatus***	Alkaloids	Shows antifungal activities against postharvest pathogens	Li et al., 2012
***Trichoderma polyalthiae***	Violaceol I and Violaceol II	Showed antimicrobial activities	Nuankeaw et al., 2020
***Streptomyces* sp.**	Enduspeptide B, neomaclafungins A-I	Strong antifungal activities	Jakubiec-Krzesniak et al., 2018
***Streptosporangium oxazolinicum K07-0460***	Polyketides	Antibacterial activities against *Xanthomonas* sp.	Matsumoto and Takahashi, 2017
***Xylariales* sp.**	α-pyrone derivatives	Antifungal activities against *Botrytis cinerea, Fusarium oxysporum* and *Alternaria* sp.	Rustamova et al., 2020
***Alternaria* sp.**	Alternarilactone-A	Antifungal activities against *Verticillium cinnabarium* and *Gaeumannomyces graminis*	Rustamova et al., 2020

Though the potential of bioactive secondary metabolites is enormous in postharvest disease control of fruits, the low quantity produced, *in planta* pressure, and influence of the culture conditions are some of the factors that need optimization.

### Mycoparasitism and production of lytic enzymes

One of the essential mechanisms employed by endophytic fungi against pathogenic fungi is mycoparasitism by the production of cell wall-degrading enzymes and direct parasitism. The lytic enzymes, including glucanase, chitinase, and cellulose produced by endophytes, can degrade the pathogenic cell wall. For example, *Talaromyces acidophilus* a fungal strain AUN-1 emerged as a novel mycoparasite of postharvest pathogen *B. cinerea* by producing lytic enzyme chitinase, lipase, and protease (Abdel-Rahim and Abo-Elyousr, 2018). Endophytic fungus *Choiromyces aboriginum* inhibited postharvest pathogen *Pythium* sp. by producing β-1,3-glucanases and degraded the pathogenic cytoplasm coiling around the hyphae (Cao et al., 2009). In the same sense, plant beneficial fungus *Trichoderma* spp. can inhibit the growth of several pathogens through parasitism, for example, a *Trichoderma* sp. strain inhibited the fungal pathogen *F. oxysporum* by producing a lytic enzyme and coiling around the pathogenic fungal hyphae (Rajani et al., 2021).

Some bacterial strains are also prolific producers of lytic enzymes, making them suitable candidates for postharvest disease management, though endophytes specifically have not been explored much. For example, endophytic *Bacillus* sp. are known to produce β-1,3-glucanase, chitinase, and protease, which can disrupt fungal cell walls (Carmona-Hernandez et al., 2019). The hydrolytic enzymes produced by *B. subtilis* 739 caused the lysis of phytopathogenic fungi *A. alternata*, *B. sorokiniana*, *F. culmorum*, and *R. solani*. The cocktail of cold-adapted lytic enzymes produced by archaea and cold-adapted bacteria has also shown their potential against antagonistic fungal pathogens (de Oliveira et al., 2020), which provides an excellent opportunity to explore endophytes from extreme conditions.

### Production of endotoxins and lipopolysaccharides

Endophytes are being developed as prolific producers of LPSs of several lengths of fatty acids. For example, phengicines and iturins produced by *B. subtilis* GA1 inhibited the growth of *B. cinerea* in apple fruits (Toure et al., 2004). Thus, the optimized media conditions for synthesizing LPSs from endophytes can pave a sustainable path for the biological control of postharvest fruit diseases. The toxin Leu7-surfactin was produced from the endophytic bacterium *Bacillus mojavensis* RRC 101 against antagonistic fungus *Fusarium verticillioides* (Snook et al., 2009). Several mycotoxins produced by endophytic fungi can also be explored for their efficacy against the antagonistic pathogens to control postharvest disease, though their safety also needs to be analyzed thoroughly (Lacava and Azevedo, 2013).

### Modulating the redox homeostasis of harvested fruits and pathogens

Many postharvest pathogens overcome the fruit defense system by manipulating their redox potential. For example, *Penicillium digitatum*, the causative agent of green mold in citrus fruits, produces catalase that decomposes hydrogen peroxide to establish an infection (Macarisin et al., 2007). Endophytes provide oxidative stress protection to plants (Hamilton et al., 2012; White et al., 2019). However, their role in modulating stress in postharvest disease management is not much explored. Endophytes help plants combat biotic stress by lowering lipid peroxidation and accumulation of proline (Spadaro and Droby, 2016). As an example, endophytic fungus *Paraburkholderia phytofirmans* strain PsJN increased the expression of genes involved in reactive oxygen species (ROS)-scavenging pathways, resulting in detoxification of ROS and modulating the signaling pathways (Pacifico et al., 2019). The plant–pathogen and endophytic relation has been documented well in literature, but the research on the role of endophytes in modulating redox homeostasis of stored fruits needs special attention.

### Quorum sensing and biofilm formation and disruption by endophytes

Bacterial endophytes, including *Bacillus* spp. and *Pseudomonas* spp., are known to colonize exposed fruit areas by quorum sensing (QS) and biofilm formation. The ability of endophytic bacteria to secrete small molecules such as tyrosol, farnesol, and phenethyl alcohol to regulate colonization helps them outnumber the pathogenic microbes in the competition for space and nutrients (Carmona-Hernandez et al., 2019). Recently, endophytes were also found to produce anti-QS molecules, which can help combat the biofilm established by pathogenic bacteria on fruit surfaces. For example, endophytic fungi *Fusarium graminearum* and *Lasiodiplodia* sp. isolated from the plant *Ventilago madraspatana* produced secondary metabolites with anti-QS potential (Mookherjee et al., 2017). Furthermore, the isolated fungi produced QS inhibitors that were quantified spectrophotometrically by their ability to inhibit the production of violacein in wild and mutants of *Chromobacterim violaceum* (Rajesh and Rai, 2013). Whether it is biofilm formation or the production of anti-QS molecules by endophytes, both properties can be exploited in postharvest disease management in fruits, as this field of research remains unexplored.

### Modulation and synthesis of phytohormones

Endophytic microbes can synthesize phytohormones, including auxin, gibberellins, cytokines, ethylene, nitric oxide, and 1-aminocyclopropane-1-carboxylic acid (ACC) deaminase, which provide additional immunity to postharvested plants to cope up with biotic and abiotic stresses (Ali et al., 2017). The increased phytohormone synthesis helps to overcome the stress-induced wilting. Not only are the endophytes capable of synthesizing plant hormones themselves, but they can also modulate the plant–hormone metabolic pathways for enhanced stress tolerance. For example, the interaction of endophytic fungus *Piriformospora indica* in the synthesis of auxins, cytokinin, gibberellins, abscisic acid, ethylene, salicylic acid (SA), jasmonates, and brassinosteroids resulted in better efficiency of stress tolerance in higher plants (Xu et al., 2018).

### Induction of disease resistance in fruits

**Figure 3:**
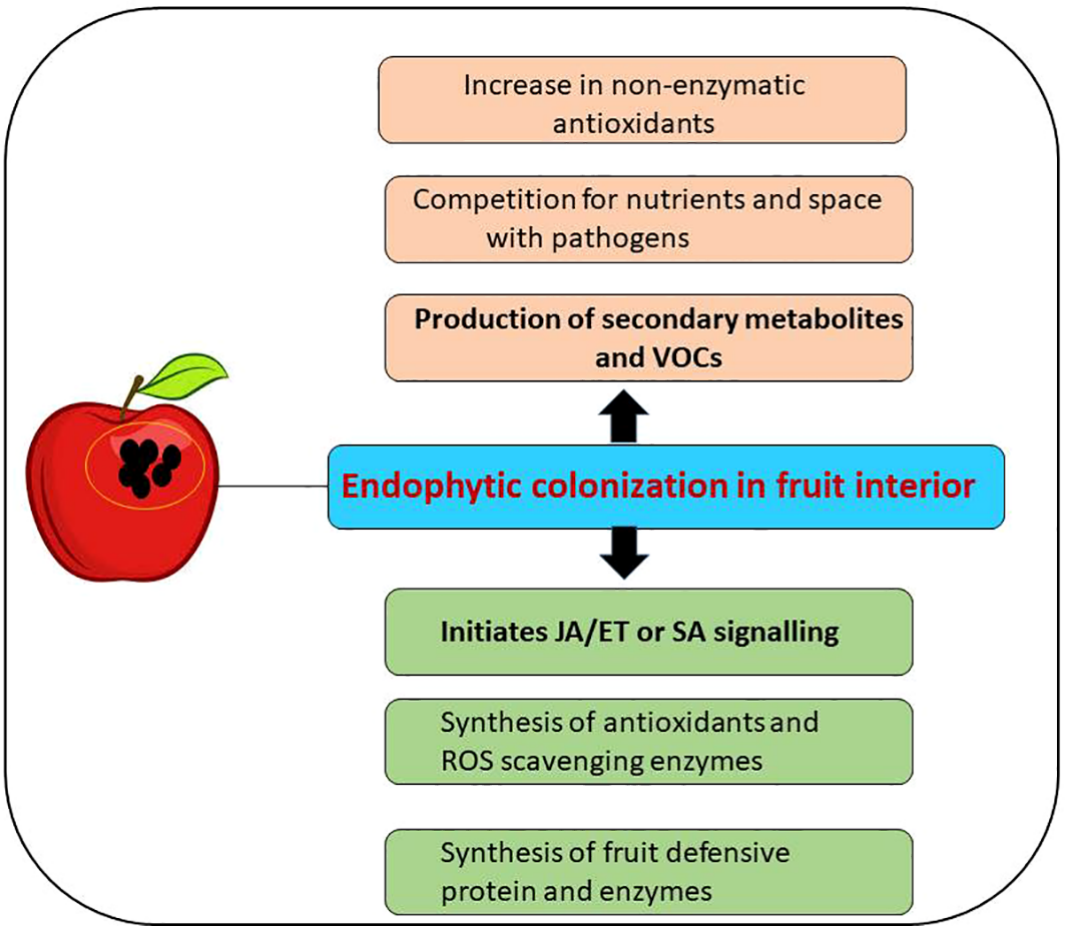
Activation of induced systemic resistance (ISR) signaling pathway and production of bioactive secondary metabolites after colonization of endophytes in the host (postharvested fruits).

In response to a pathogenic attack, plants develop two kinds of disease resistance mechanisms: 1) systemic acquired response (SAR) and 2) induced systemic resistance (ISR). Many endophytic microbes have been known to elicit ISR, thereby providing solid immunity against biotic stress (Pacifico et al., 2019). Endophytes activate ISR pathways by synthesizing pathogen-related proteins, enhancing the synthesis of phenolic compounds, and activating signaling pathways by jasmonate/SA and ethylene (Jacob et al., 2020) (**Figure 3**).

The endophytic bacterial strain *Pseudomonas putida* MGY2 was able to control anthracnose caused by *C. gloeosporioides* in harvested papaya fruit (Shi et al., 2011). It was found that the endophyte induced ISR by increasing the gene expression of phenylalanine ammonia-lyase (PAL), catalase (CAT), and peroxidase (POD), increasing the phenolic content and decreasing the production of ethylene. The same group demonstrated the control of *Phytophthora nicotianae* disease in papaya fruits by induction of the pathogenesis-related protein 1 gene (PR1) and non-expression of PR1 gene (NPR1) after inoculation of *P. putida* MGP1 strain (Shi et al., 2013). Louarn et al. (2013) demonstrated a significant change in the endophytic community in organically and conventionally grown carrots. Endophytic *Bacillus amyloliquefaciens* YTB1407 strain elicited ISR by activating the expression of SA-responsive PR1 gene, thus inhibiting pathogenic fungus *Fusarium solani*. The literature is insufficient regarding the elicitation of molecular responses of fruits in postharvest conditions. Furthermore, in-depth mechanistic studies are required to understand the disease resistance of fruits after endophytic microbe application.

### Modulating the native microbiota and ecological effects

The endophytic microbial population modulates the native microbiota of fruits, roots, leaves, and soil, promoting a sustainable crop production system. Therefore, it is of great economic relevance (Sturz et al., 2010; Baghel et al., 2020). However, its interference with the native population of harvested fruit microbiota is still waiting to be explored. Endophytes bear the potential to shift the native bacterial population toward favorable conditions for plant growth and stress amelioration (Baghel et al., 2020). It has been found that healthy fruits tend to have a diverse microbial community, whereas diseased fruits have a limited microbial growth dominated by pathogen microorganisms (Huang et al., 2021). In their study, Diskin et al. (2017) found that colonization of endophytic communities was much less prevalent in mango fruits suffering from stem-end rot disease than that in their healthier counterpart. By utilizing multiple mechanisms, including parasitism, production of bioactive compounds, lytic enzymes, and siderophores against postharvest pathogens, endophytes can modulate the native microbiota of the harvested fruits to increase their resistance against biotic stresses.

### Controlling mycotoxins

Mycotoxins are a major cause of qualitative and quantitative loss in stored fruits. Deoxynivalenol, alternariol, aflatoxin, and patulin, produced by antagonistic fungi, can impact fruit and human health negatively (Bartholomew et al., 2021). Many endophytes and their secondary metabolites have shown the effectiveness of controlling mycotoxins *in vitro* and *in planta* (Abdallah et al., 2018) in maize and other crops, although studies on their impact on postharvested fruits are limited. Sarrocco and Vannacci (2018) emphasized preharvest application of endophytes for controlling postharvest damage caused by mycotoxins. The VOCs produced by endophytic fungi can be incorporated in edible biofilms or can be an ingredient during packaging to effectively control mycotoxins in store fruits (Mari et al., 2016).

As biocontrol strategies usually rely on a single or mixture of antagonists, endophytic microbial strains have been suggested as antagonistic microorganisms against various diseases in various crops. The additional effect of endophytic microbiota as BCAs is the phytohormone synthesis, metabolites, and nutrients utilized for growth promotion and stress management in host plants (Lodewyckx et al., 2002; Suhandono et al., 2016).

In the recent past, various BCAs, including bacteria, yeast, and fungi, have been frequently applied for effective management of postharvest pathogens, while practices with endophytes are very limited. Endophytes’ properties appear superior to those of epiphytic microorganisms due to their better colonization and tolerance potential against various biotic and abiotic stresses (Shi et al., 2010). In recent years, several pieces of literature regarding utilizing the endophytic microbiome for screening BCAs against postharvest pathogens have been reported. Shimizu et al. (2009) reported on the endophytic actinomycete *Streptomyces* sp., which showed effective biocontrol potential against the pathogen *Colletotrichum orbiculare*, the causal agent of anthracnose disease in cucumber. Similarly, Shi et al. (2010) reported on *P. putida* biovar isolated from the pericarp of papaya with strong colonization potential and showed potent inhibition against several pathogens.

Additionally, the strain effectively inhibits the growth of *P. nicotianae* just after a short period of treatment. Lai et al. (2012) screened the endophytic strain *Paenibacillus polymyxa* isolated from the root tissue of *Sophora tonkinensis* and showed antagonistic potential against *P. digitatum*, one of the most devastating pathogens causing postharvest diseases in citrus fruit. The application of endophytic strains effectively reduces postharvest decay by inhibiting conidia germination in a fungal cell suspension. Additionally, the unwashed cell suspension of the strain was found to be more effective than the washed cell suspension and culture filtrate in the *in vivo* trials.

Ji et al. (2008) isolated 45 endophytic bacterial strains from the mulberry leaves (*Morus alba* L.) and reported the strong inhibitory potential of *B. subtilis* Lu144 against *Ralstonia solanacearum*, the causal agent of bacterial wilt of mulberry fruits. Furthermore, Furuya et al. (2011) utilized the strain *B. subtilis* KS1 isolated from the skin part of grape berry and applied it as a potential antagonistic agent against fungal grapevine diseases. *In vitro* screening showed that the strain effectively suppressed the growth of *B. cinerea* and *C. gloeosporioides.* Furthermore, after applications in the vineyards, the strains significantly reduce the incidence of downy mildew from the leaves and skin of the berry. Chen et al. (2016) screened the *B. amyloliquefaciens* PG12 strain isolated from apple fruits as a potential BCA against apple ring rot disease. The strain significantly suppressed the *Botryosphaeria dothidea* growth during *in vivo* and *in vitro* screening and showed a potent antagonistic effect against different fungal pathogens. Madbouly et al. (2020) evaluated the biocontrol potential of endophytic yeast strains *Schwanniomyces vanrijiae*, *Galactomyces geotrichum*, *Pichia kudriavzevii*, isolated from apple fruits, against the pathogen *Monilinia fructigena*, the causal agent of apple fruit brown rot of golden delicious apples. During *in vitro* test analysis, all three endophytic yeast strains showed inhibitory potential against *M. fructigena* and significantly inhibited conidial germination by 67.6%–89.2%. In the last few years, rapid enhancement can be seen in the use of endophytic microorganisms in postharvest disease management in fruits. However, still, most of the experiments are limited to the laboratory scale. Furthermore, we need to study how the fruit microbiome affects the fruit’s physiology and disease resistance and how the fruit-associated microbial communities shifted during the postharvest stages and after applying BCAs.

## Commercial upscale production and hurdles ahead

Antagonistic endophytic application against postharvest diseases, especially in fruits, has emerged as a new generation of pesticides. Though the mechanisms are still to be deciphered completely, many endophytes have paved their path to commercial applications. *B. subtilis* strain B-3 has been patented, and pilot experiments have been conducted against the peach brown rot disease. It was observed that after the application of the endophyte in either powder or paste form, it was as effective as traditional pesticide benomyl in Clemson, SC, USA (Pusey et al., 1988). Products based on *B. subtilis* QST713 with the trade name Serenade™ are produced commercially by AgraQuest Inc., USA, against powdery mildew, brown rot, and late blight of apple, pear, and grapes (Punjia et al., 2016). Multiple formulations in many countries with trade names, including Candifruit™, Shemer™, and Boni-protect™, have been successfully used against postharvest pathogens (Fenta et al., 2019). The endophytes, a new concept, have to face many hurdles for their successful commercialization. In addition to the agricultural giants such as Dupont, Monsanto, and Bayer, many small startup companies such as Indigo and NewLeaf Symbiotics have entered the microbial domain with promising contributions. The following hurdles need to be overcome to achieve economically and sustained commercial-scale production of antagonistic endophytes or their products.

### Increased shelf life and multiple stress-tolerant endophytic microbes

In the niche of postharvest fruits, endophytes have to overcome several biotic and abiotic stresses (Diskin et al., 2017). For the successful application and upscale production of antagonistic endophytes against postharvest diseases of fruits, the endophytes must be stress-tolerant to prolong their shelf life and sustain antipathogenic activities. Many stress-tolerant endophytic microbes are already studied for plant growth promotion in adverse conditions (Giauque et al., 2019; Singh et al., 2022). Furthermore, the synergistic application of endophytes can also help increase the shelf life of endophytes in their battle against postharvest pathogens (Huang et al., 2021). Therefore, exhaustive screening of stress-tolerant endophytes and their *in vitro* and *in vivo* stress amelioration potential should be conducted for the endophytes to go from lab to field.

Some endophytes are deeply associated with their host for stress tolerance and the production of the desired natural products (Khare et al., 2018). Therefore, their ability to cope up with the stress condition in the absence of their host plants and the niche of postharvest fruits should also be assessed before their commercialization.

### Optimizing the modes of endophyte application

The modes of application of endophytes to the surface of postharvest fruits also play a crucial role in plant disease management and increasing the shelf life of the endophytes. Therefore, the application of endophytes on fruit surfaces should be optimized on a case-by-case basis. Generally, the formulations are applied as liquid or powder/paste formulations. Though the dry form provides a longer shelf life, it can cause a loss of viability of microbes through repeated rehydration-dehydration processes (Kumari et al., 2020a, b). Many rehydration agents, including whey proteins and maltodextrins, have been suggested to coat dry formulations (Martin et al., 2017). For sustained release of endophytes, their secondary metabolites, and VOCs, nanoencapsulation of the products and nanoemulsions can also be studied (Pandey et al., 2020). Recently, Ghazy et al. (2021) studied the role of anise extract oil nanoemulsion against different postharvest antagonistic bacteria for their sustained release. A combination of SA with endophytic *B. subtilis* was used to treat postharvest diseases by *F. oxysporum* and *P. infestans* (Lastochkina et al., 2020). Preharvest and postharvest modes of endophytic application should also be considered for their antagonistic application. For the upscale production of endophytes as postharvest disease management in fruits, the mode of application is an important parameter, whose optimization should be carried out in detail.

### Sustained release and cost-effective production of microbial metabolites

The commercialization of secondary metabolites and VOCs derived from endophytes faces hurdles in sustainable release and economic upscale production. Media optimization, selection of potent microbial strains, and metabolic engineering are some of the parameters that can be employed (Sah et al., 2020; Kamat et al., 2020; Taritla et al., 2021) for the sustained production of desired antimicrobial secondary metabolites from endophytes. The addition of some of the precursors from the host system has also been studied during media optimization for continuous upscale production of the antimicrobial metabolites from endophytes during the fermentation process.

The second hurdle faced during their commercialization includes the hydrophobicity of natural products. To overcome the solubility issue, several solutions, including their encapsulation in non-toxic and biodegradable polymers, have been proposed (Soh and Lee, 2019), which provide solubility and the slow release of the active ingredient. Chitosan, carrageenan, starch, and alginate nanopolymers have been used to encapsulate natural products, including polyphenols, alkaloids, and terpenoids with increased water solubility and bioactivity (Detsi et al., 2020).

### Overcoming the *in planta* pressure for survival and stress amelioration

The biggest hurdle in successfully applying endophytic microbes in the fruit microbiome is overcoming their host pressure. Endophytes have always lived as symbionts with their host, sharing many physical and chemical attributes with their host plants (Spadaro and Droby, 2016). Several hypotheses, including the defensive mutualism hypothesis, xenohormesis hypothesis, and trait-specific endophytic infallibility (TSEI) hypothesis, have been shared among the research community to describe the co-evolution of the host and the endophytes (Kusari et al., 2015; Pathak et al., 2022). Their isolation and survival without their hosts may alter their growth cycle and physiological performance in the competition of the new fruit microbiome. The question of replacement dynamics with the preexisting microbiome of fruits is always relevant while introducing a new endophytic strain. The mode of application and the growth and production of secondary metabolites *in vitro* should be monitored before their *in vivo* application in postharvested fruit microbiomes.

### Genome mining and metagenomics

Getting the superior strains of endophytes required digging deep into the unexplored wealth of endophytes and exploring the biosynthetic pathways to synthesize beneficial secondary metabolites, siderophores, and phytohormones. To bypass the tedious process of endophyte isolation and screening for postharvest disease management, genome mining and metagenomic studies can be performed to select the right strain economically (Kusari et al., 2015). For example, genome mining of the endophytic fungus *Penicillium dangeardii* revealed a cluster of 43 biosynthetic genes demonstrating their strong ability to synthesize secondary metabolites (Wei et al., 2021) exploited in postharvest disease management. Thus, genome mining and metagenomics can provide better endophytic strains that can be commercially produced for the desired secondary metabolites.

### Change in policymaking and awareness regarding the use of antagonistic endophytes

The most critical parameter for introducing endophytes as substitutes for conventional pesticides in postharvest disease management is to increase the awareness of the end-users and people involved in the distribution chain. Therefore, outreach programs and workshops related to these new ideas should constantly be organized to bring awareness and benefits of using endophyte-based biopesticides.

Any effort is not fruitful without governments, policymaking, and funding agencies to implement new technologies in agri-business sectors. Earlier, the Department of Biotechnology (DBT), India, launched the National Biocontrol Network Programme (NBNP) to popularize and commercialize more than 30 biopesticides (Kumari et al., 2020a, b). Similar programs should be launched and funded to popularize financial, most effective, and eco-friendly products for managing postharvest diseases of fruits.

### Safety of endophytes and their secondary metabolites for consumers and the environment

Endophytes, a new aspect of BCAs in postharvest disease management in fruits, need thorough scrutiny regarding their safety for consumers and the environment. Endophytes themselves or their products should not be opportunistic pathogens or should not pose any harm to the environment. Unfortunately, many of the earlier studied rhizobacteria or their secondary metabolites have acted as opportunistic human pathogens or environmental contaminants in certain conditions (Keswani et al., 2019). To avoid similar conditions with the endophytes, their safety in animal models and their effect on the environment due to higher dosage should also be assessed.

## Conclusion

Endophytic microorganisms can colonize different organ tissues of the host plant and interact in multiple ways to regulate physiological and metabolic pathways, which can further be utilized in the effective management of postharvest diseases. Endophytic bacterial, actinomycetes, and fungal strains have been broadly utilized as BCAs against various plant pathogens during preharvest and postharvest stages. Currently, it is estimated that approximately 30% of the total fruit production is lost annually due to various diseases. Therefore, the potential colonization efficacy of endophytes is a crucial characteristic for disease management.

In addition, next-generation omics may be applied to identify the gene(s) responsible for disease management. Thus, during the application, consortia of mixed microbial agents (bacteria-bacteria; bacteria-fungus; fungus-fungus) showed a practical approach in disease management, but the survival and better adaptability of both strains together are reasons for further investigation, particularly under diverse environmental conditions. Endophytes have reported multiple mechanisms that are used to inhibit pathogenic growth and increase fruit health. Though there are numerous examples of successful bioformulations of microbial endophytic strains capable of controlling the pathogenicity of the pest or pathogens during preharvest conditions, their application in postharvest pathogen control is in the nascent stage. Further application of endophytic microbiome can further reduce, or at some point will eliminate, the harmful dependence on chemical pesticides and fungicides in postharvest disease management.

## Author contributions

MK and AK designed the study. MK, KQ ,SS, VS, KS, and AK wrote the manuscript. KQ and MJ acquired funding. KQ, MJ, JW, GS, and GP reviewed and provided valuable feedback to this study. All the authors contributed to the article and agreed to the published version of the manuscript.

## Funding

The research is financially supported by King Abdullah University of Science and Technology, Thuwal, Jeddah, Saudi Arabia.

## Acknowledgments

The authors are thankful to the Agriculture Research Organization for providing lab facilities.

## Conflict of interest

The authors declare that the research was conducted in the absence of any commercial or financial relationships that could be construed as a potential conflict of interest.

The reviewer SP declared a shared affiliation with the author SS to the handling editor at the time of review.

## Publisher’s note

All claims expressed in this article are solely those of the authors and do not necessarily represent those of their affiliated organizations, or those of the publisher, the editors and the reviewers. Any product that may be evaluated in this article, or claim that may be made by its manufacturer, is not guaranteed or endorsed by the publisher.
